# Quantifying and Adjusting for Disease Misclassification Due to Loss to Follow-Up in Historical Cohort Mortality Studies

**DOI:** 10.3390/ijerph121012834

**Published:** 2015-10-15

**Authors:** Laura L. F. Scott, George Maldonado

**Affiliations:** Division of Environmental Health Sciences, University of Minnesota School of Public Health, Minneapolis, MN 55455, USA; E-Mail: GMPhD@umn.edu

**Keywords:** probabilistic bias analysis, Monte Carlo, disease misclassification, loss to follow-up, historical cohort mortality

## Abstract

The purpose of this analysis was to quantify and adjust for disease misclassification from loss to follow-up in a historical cohort mortality study of workers where exposure was categorized as a multi-level variable. Disease classification parameters were defined using 2008 mortality data for the New Zealand population and the proportions of known deaths observed for the cohort. The probability distributions for each classification parameter were constructed to account for potential differences in mortality due to exposure status, gender, and ethnicity. Probabilistic uncertainty analysis (bias analysis), which uses Monte Carlo techniques, was then used to sample each parameter distribution 50,000 times, calculating adjusted odds ratios (*OR_DM-LTF_*) that compared the mortality of workers with the highest cumulative exposure to those that were considered never-exposed. The geometric mean *OR_DM-LTF_* ranged between 1.65 (certainty interval (CI): 0.50–3.88) and 3.33 (CI: 1.21–10.48), and the geometric mean of the disease-misclassification error factor (*ε_DM-LTF_*), which is the ratio of the observed odds ratio to the adjusted odds ratio, had a range of 0.91 (CI: 0.29–2.52) to 1.85 (CI: 0.78–6.07). Only when workers in the highest exposure category were more likely than those never-exposed to be misclassified as non-cases did the *OR_DM-LTF_* frequency distributions shift further away from the null. The application of uncertainty analysis to historical cohort mortality studies with multi-level exposures can provide valuable insight into the magnitude and direction of study error resulting from losses to follow-up.

## 1. Introduction

Epidemiologists have several means available with which to evaluate the exposure-disease relationship in occupational settings. One of the most frequently used methods is the historical cohort mortality study [1,2]. In general, this type of study offers several benefits in that it typically requires less time to complete, is inexpensive compared to other types of studies, and is well-suited for evaluating multiple outcomes and occurrences of rare diseases. However, historical cohort studies are also vulnerable to loss to follow-up, with one method of addressing this being to withdraw lost individuals from the analysis at the time of loss [1,3,4].

Detailed methods for conducting bias analysis (probabilistic uncertainty analysis) of epidemiologic studies have been described by a number of researchers and methodologists [5,6,7,8,9,10,11,12]. Historical cohort mortality studies, however, provide distinct challenges for quantifying study error. First, these types of studies commonly use person-time as the denominator of disease frequency measures; yet bias analysis methods for adjusting an estimate with a person-time denominator have not been described in the peer-reviewed literature. Consequently, to conduct a bias analysis of a cohort mortality study one must use a measure which does not rely on person-time (*i.e.*, odds ratio, incidence proportion ratio, *etc.*). Although using one of these measures would seem to be a simple solution, it introduces another issue unique to historical cohort mortality studies: disease misclassification due to loss to follow-up, which can result from counting those lost to follow-up who have died as alive at the end of a study. Bias analysis methods to account for this type of disease misclassification have not been previously described. Here, we describe such a method and illustrate how it can be applied to a historical cohort mortality study of New Zealand trichlorophenol workers.

## 2. Methods

### 2.1. Error Term for Disease Misclassification Due to Losses

In 2008, Maldonado [12] detailed the mathematical relationship between a causal relative risk, an observed relative risk, and error terms for study bias. We have provided a modification of this relationship (Equation (1)), where *OR_DM- LTF_* is the odds ratio adjusted for disease misclassification due to loss to follow-up, *OR_observed_* is the observed crude odds ratio, and *ε_i_* are the terms which quantify the systematic error in a study. Because in this manuscript only one error is being evaluated, the denominator has been simplified to ε*_DM-LTF_*, the error term for disease misclassification due to loss to follow-up. *ε_DM-LTF_* is calculated by taking the ratio of the observed odds ratio to the adjusted odds ratio.

(1) $$OR_{DM-LTF}=\frac{OR_{observed}}{\prod_{i=1}^{n}\varepsilon_{i}}=\frac{OR_{observed}}{\varepsilon_{DM-LTF}}$$

### 2.2. Crude Odds Ratio

Using the mortality data described by McBride *et al.* [13], we calculated a crude odds ratio for the association between ischemic heart disease (IHD) mortality and exposure to 2,3,7,8-tetrachlorodibenzo-*p*-dioxin (TCDD). The odds of IHD death for the group with the highest TCDD exposure was 14/148 = 0.0946, and the odds of IHD death for “never-exposed” workers was 14/451 = 0.0310, giving an observed odds ratio of 3.05 and a 95% confidence interval of 1.42–6.54 (Table 1).

**Table 1:** Cell counts used to estimate the crude odds ratio and 95% confidence limits for the association between occupational TCDD exposure and ischemic heart disease using data reported by McBride *et al.* [13].

Outcome	TCDD Exposure		
	≥2085.8 ppt-mo	0–2085.7 ppt-mo	Never-Exposed
IHD Cases	14	47	14
Non-cases	148	925	451
Alive	112	826	414
Deceased **^a^**	36	99	37

**^a^** From causes of death other than IHD.

### 2.3. Number of All-Cause Deaths among Losses to Follow-up

**Figure 1:**
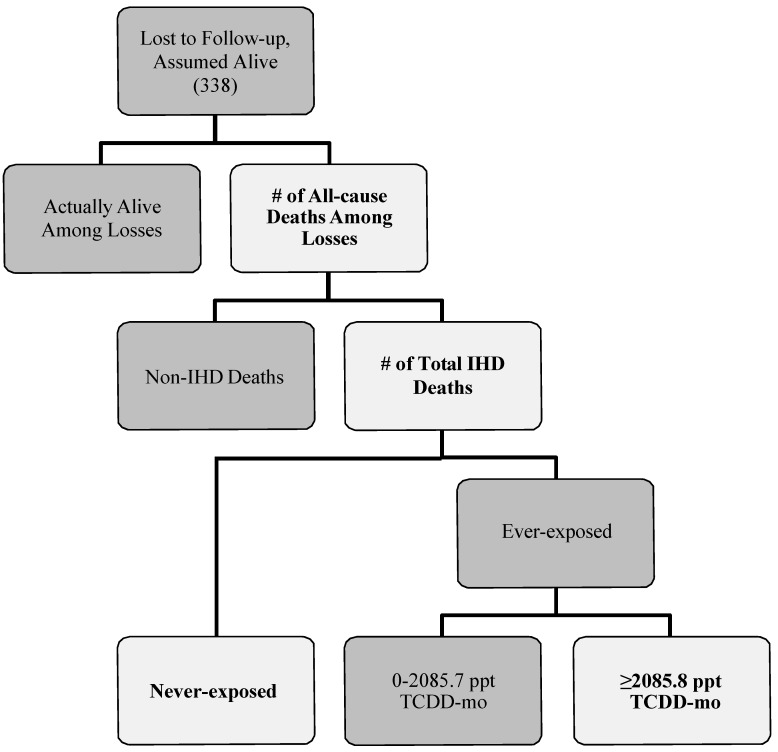
Flow diagram describing how losses to follow-up in Mcbride *et al.* [13] could result in outcome misclassification. Lighter shapes with bolded text indicate the parameters that were specified in our bias analysis.

To estimate the number of workers lost that could have died from IHD for each exposure category, we used a multi-step process (Figure 1). First, we defined a probability distribution for the total number of those lost to follow-up that may have died from any cause for all exposure levels combined. A total of 338 individuals (~21% of the cohort) were lost to follow-up in the cohort mortality study published by McBride and colleagues [13]. We assumed that anywhere from zero to 338 individuals might have died from any cause. Therefore, the minimum and maximum of the probability distribution were set to zero and 338, respectively. We specified the peak of this probability distribution by using the proportions of known deaths observed for the cohort. These ranged from 11.0%, the observed proportion of all deaths in the never-exposed category, to 30.9%, the observed proportion of all deaths in the highest exposure category. The peak number of all-cause deaths for this probability distribution was estimated by multiplying each proportion by the total number of individuals lost. More specifically, 30.9% of 338 provided a peak value of 104 (Scenarios 1–4 in Table 2) and 11.0% of 338 provided a peak value of 37 (Scenarios 5–8 in Table 2). We chose a negative binomial distribution—a discrete distribution with more flexibility than the Poisson distribution for providing the desired shape of the probability distribution—with lower and upper truncation points of 0 and 338, respectively, to describe the spread of the number of all-cause deaths in those workers that were lost. These minimum, maximum and peak values determined the probability and shape input of all-cause deaths for each bias-analysis scenario.

**Table 2:** Bias-analysis scenarios: description of probability distributions for classification parameters used to estimate the number of workers lost to follow-up that could have died from IHD and corresponding geometric mean errors (*ε_DM-LTF_*), adjusted odds ratios (*OR_DM-LTF_*) and 95% bias-analysis certainty intervals.

Scenario	Total All-Cause Deaths	Total IHD Deaths	IHD Deaths by Exposure Status			*ε_DM-LTF_*		*OR_DM-LTF_*	
	Distribution (Parameters)	Distribution (Parameters)	Direction of Misclassification	Distribution (Parameters)-Never-exposed	Distribution (Parameters)-≥2085.8 ppt TCDD-mo	GM	95% Certainty Interval	GM	95% Certainty Interval
**1**	Negative Binomial **^a^** (0.02, 3)	BetaPERT **^b^** (0, 0.204 **^c^** × AD, AD)	Differential A **^d^**	BetaPERT (0, 3/4 × ID, ID)	BetaPERT (0, 1/2 × IDE, IDE) **^e^**	1.85	0.78–6.07	1.65	0.50–3.88
**3**	Negative Binomial **^a^** (0.02, 3)	BetaPERT (0, 0.139 **^c^** × AD, AD)	Differential A	BetaPERT (0, 3/4 × ID, ID)	BetaPERT (0, 1/2 × IDE, IDE)	1.62	0.74–4.93	1.88	0.62–4.11
**5**	Negative Binomial **^f^** (0.027, 2)	BetaPERT (0, 0.204 × AD, AD)	Differential A	BetaPERT (0, 3/4 × ID, ID)	BetaPERT (0, 1/2 × IDE, IDE)	1.50	0.87–3.92	2.03	0.78–3.51
**7**	Negative Binomial **^f^** (0.027, 2)	BetaPERT (0, 0.139 × AD, AD)	Differential A	BetaPERT (0, 3/4 × ID, ID)	BetaPERT (0, 1/2 × IDE, IDE)	1.43	0.87–3.65	2.13	0.83–3.50
									
**2**	Negative Binomial **^a^** (0.02, 3)	BetaPERT (0, 0.204 × AD, AD)	Differential B **^g^**	BetaPERT (0, 1/4 × ID, ID)	BetaPERT (0, 1/2 × IDE, IDE)	0.91	0.29–2.52	3.33	1.21–10.48
**4**	Negative Binomial **^a^** (0.02, 3)	BetaPERT (0, 0.139 × AD, AD)	Differential B	BetaPERT (0, 1/4 × ID, ID)	BetaPERT (0, 1/2 × IDE, IDE)	0.92	0.32–2.37	3.31	1.29–9.65
**6**	Negative Binomial **^f^** (0.027, 2)	BetaPERT (0, 0.204 × AD, AD)	Differential B	BetaPERT (0, 1/4 × ID, ID)	BetaPERT (0, 1/2 × IDE, IDE)	0.95	0.42–1.98	3.20	1.54–7.26
**8**	Negative Binomial **^f^** (0.027, 2)	BetaPERT (0, 0.139 × AD, AD)	Differential B	BetaPERT (0, 1/4 × ID, ID)	BetaPERT (0, 1/2 × IDE, IDE)	0.96	0.46–1.86	3.18	1.64–6.64

AD, number of total all-cause deaths; ID, number of total IHD deaths; IDE, number of IHD deaths for those workers ever-exposed; **^a^** Negative binomial distribution (probability, shape)—probability and shape were determined based on minimum, likeliest and maximum counts of (0, 104, 338); **^b^** BetaPERT distribution (minimum, likeliest, maximum); **^c^** 0.204: proportion of all-cause deaths due to IHD among non-Maori males; 0.139: proportion of all-cause deaths due to IHD among Maori females; **^d^** Never-exposed more likely to be misclassified as alive than highest exposed; **^e^** The maximum value for this distribution is capped at 112, which is the number of individuals in the highest exposure group (*i.e.*, ≥2085.8 ppt-mo) that were classified as living non-cases; **^f^** Negative binomial distribution (probability, shape)—probability and shape were determined based on minimum, likeliest and maximum counts of (0, 37, 338); **^g^** Never-exposed less likely to be misclassified as alive than highest exposed.

### 2.4. Number of Total IHD Deaths among Losses to Follow-Up

Next, we used 2008 mortality data for the New Zealand population [14] to estimate the total number of deaths from IHD for all exposure levels combined. The proportion of New Zealanders who died from IHD varied by both gender and ethnicity, with the proportion of IHD deaths the highest in non-Maori males (20.4%) and the lowest in Maori females (13.9%). The BetaPERT (*i.e.*, PERT) distribution, which is derived from the beta distribution and is a smoother alternative to the triangular distribution, was specified as the probability distribution for this disease-classification parameter. We selected this distribution over the negative binomial and Poisson distributions because (1) it is considered to be ideal for modeling expert opinion of a variable [15], (2) it was much more flexible than the Poisson distribution, and (3) the maximum and likeliest values of the distribution, which were dependent on the total number of all-cause deaths selected in the first step, could easily be varied. For example, if the number of all-cause deaths was 100 for a bias-analysis simulation trial, then the distribution for the number of deaths from IHD would range from 0 to 100 with a likeliest value of 13.9, assuming 13.9% of all-cause deaths were due to IHD. Since the BetaPERT distribution is continuous and we are interested in estimating discrete counts, we used the TRUNC function in Excel to remove the decimal portion of each bias-analysis simulation trial value. The difference in the adjusted odds ratios and error terms estimated with and without use of the TRUNC function was negligible.

### 2.5. Number of IHD Deaths among Losses to Follow-Up: Never-Exposed

For the next step, we specified a probability distribution for the number of IHD deaths for the “never-exposed” group. The BetaPERT distribution, along with the TRUNC function in Excel, was also used for this parameter. The maximum and likeliest values were adjusted in a similar manner to that for the total number of IHD deaths. The maximum was set to equal the total number of IHD deaths selected in the previous bias-analysis simulation step. Since the total number that died of IHD could be categorized into one of three exposure groups (*i.e.*, “never-exposed”, “0–2085.7 ppt-mo”, “≥2085.8 ppt-mo”), we used simple fractions to determine the likeliest value of this probability distribution depending on whether IHD deaths among the “never-exposed” were (1) more likely to be misclassified as those in the highest exposure group (Differential A) or (2) less likely to be misclassified as those in the highest exposure group (Differential B). When it was assumed that the “never-exposed” were more likely to be misclassified compared to those in the highest exposure group, the likeliest value was set to equal 3/4 the total number of IHD deaths. Under the second assumption, the likeliest value was set to equal 1/4 the total number of IHD deaths. For example, if the total number of IHD deaths selected in the second step of a bias-analysis simulation trial is 24, then the likeliest values for the “never-exposed” group would be 18 and six, respectively.

### 2.6. Number of IHD Deaths among Losses to Follow-Up: ≥2085.8 ppt TCDD-mo

Last, a probability distribution for the number of IHD deaths among workers with the highest exposure (*i.e.*, ≥2085.8 ppt TCDD-mo) was specified. For this parameter, we again chose to use the BetaPERT distribution, truncating each trial value at the decimal point to obtain a whole number. Using the IF function in Excel, the distribution maximum was set to equal the number of IHD deaths for those “ever-exposed” up to 112, which is the number of individuals in the highest exposure group that were classified as living non-cases, and equal to 112 when the total number of “ever-exposed” that were potentially misclassified as alive (*i.e.*, died of IHD) was greater than 112. The likeliest value of the probability distribution for the highest exposure group was set to equal 1/2 the number of IHD deaths for the “ever-exposed” workers so that approximately 3/8 (*i.e.*, 3/4 × 1/2) and 1/8 (*i.e.*, 1/4 × 1/2) of the total number of IHD deaths would fall into the highest exposure category.

**Figure 2:**
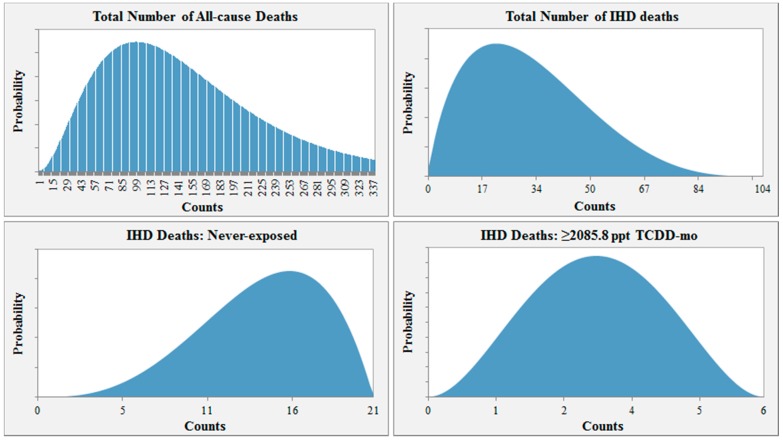
Example of parameter distribution input for Scenario 1.

For example, the probability distribution input used for Scenario 1 is shown in Figure 2. In this scenario, the distribution for the total number of all-cause deaths among those lost to follow-up ranges from 0 to 338 and peaks at 104 (30.9% of the number lost to follow-up). Assuming 104 all-cause deaths, the distribution for the total number of IHD deaths would then range from 0 to 104, with the highest probability at 21.2 or 20.4% of 104. Given 21 total IHD deaths, the likeliest number of workers misclassified as alive would then be 15 for the “never-exposed” category (3/4 × # IHD deaths = 3/4 × 21 ≈ 15). For the highest exposure group, the likeliest number of workers lost to follow-up that may have died from IHD would be three ((# total IHD deaths – # never-exposed IHD deaths) × 1/2 = (21 − 15) × 1/2 = 3).

### 2.7. Scenarios and Monte Carlo Simulation Methods

Combining the different distributions for each classification parameter resulted in eight scenarios (Table 2). The distributions of the classification parameters were sampled with Crystal Ball software [16] to generate adjusted counts of IHD cases and non-cases by exposure group, which we used to calculate adjusted odds ratios and study error. Since Crystal Ball will not allow the program to proceed when zero (or a number truncated to zero) is selected for a given classification parameter, we created a conditional action—using the IF function in Excel—whereby the program would automatically set the ensuing classification parameter values to zero anytime this occurred. For each of the eight scenarios, we conducted 50,000 trials to generate frequency distributions for *OR_DM-LTF_* and *ε_DM-LTF_* as well as 95% certainty intervals. Under specific conditions, a 95% certainty interval may approximate a 95% Bayesian posterior probability interval, such that there is a 95% chance that the true estimate for the sample population will fall within the interval [17,18,19]. This interpretation is different from that of a 95% confidence interval, which is defined as a range of values that will include the true parameter value 95% of the time.

## 3. Results

**Figure 3:**
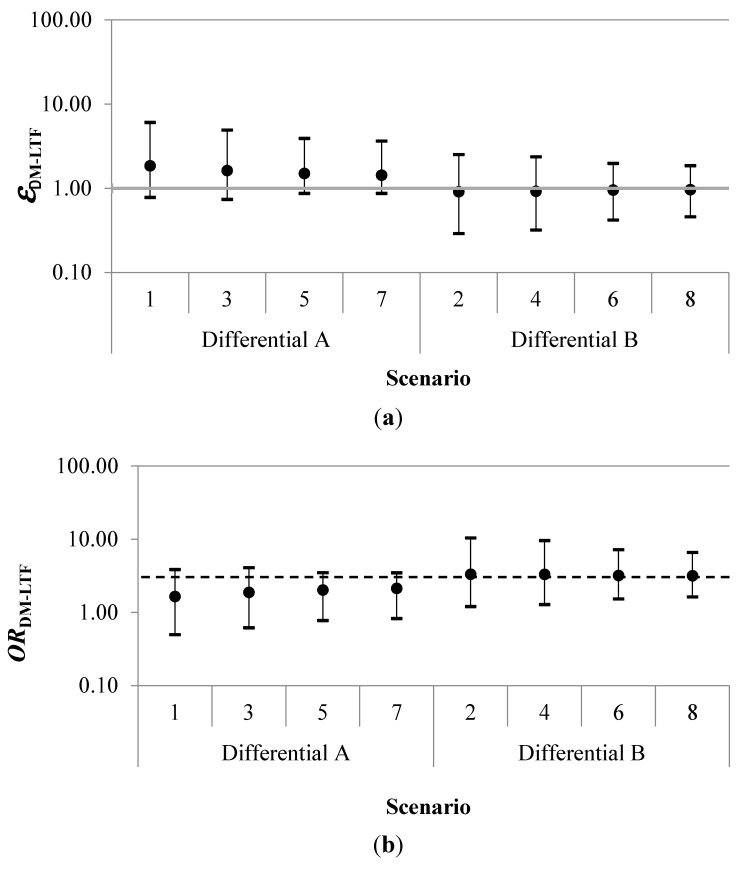
Geometric mean errors (*ε_DM-LTF_*) (**a**), adjusted odds ratios (*OR_DM-LTF_*) (**b**) and 95% certainty intervals by scenario. The dashed horizontal black line in (**b**) indicates the crude odds ratio (*OR_observed_*) of 3.05. In the Differential A scenarios, the “never-exposed” were more likely to be misclassified as alive than the highest exposed. In the Differential B scenarios, the “never-exposed” were less likely to be misclassified as alive than the highest exposed.

Results for each simulation of the probabilistic uncertainty analysis are summarized in Table 2 and Figure 3. The geometric mean of the error term for disease misclassification due to loss to follow-up (*ε_DM-LTF_*) had a range of 0.91 to 1.85. The geometric mean adjusted odds ratio (*OR_DM-LTF_*) ranged between 1.65 and 3.33. Estimated certainty intervals (CI) for the geometric mean *OR_DM-LTF_* excluded the null for all four scenarios in which those categorized as “never-exposed” were less likely to be misclassified as alive than workers in the highest exposure category.

**Figure 4:**
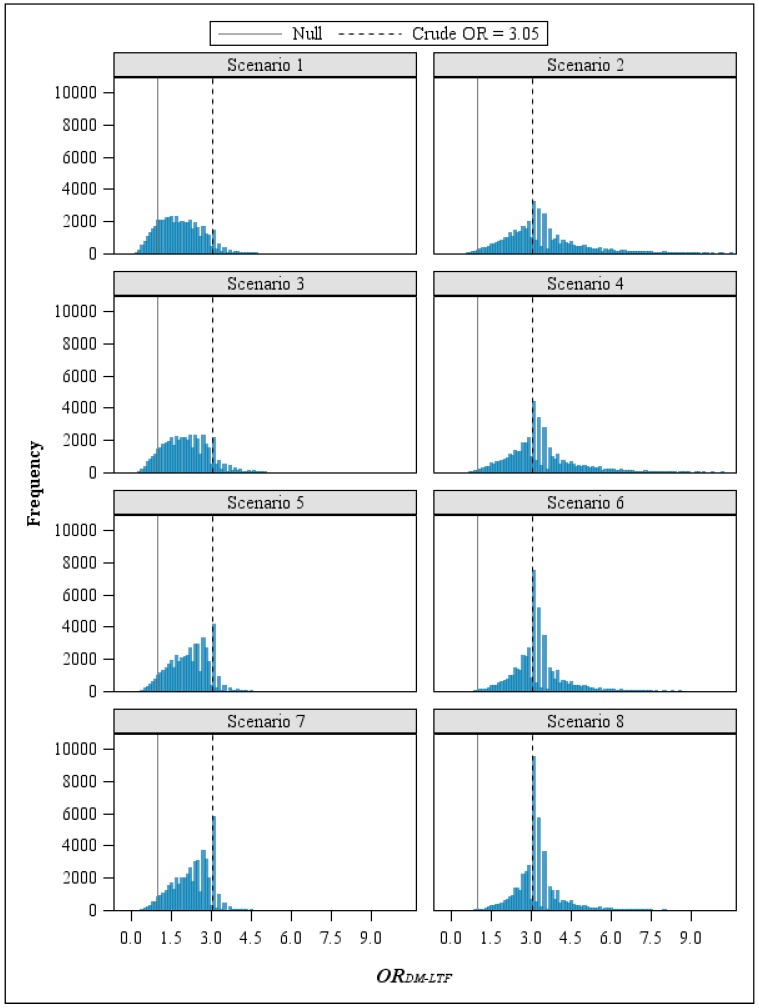
Frequency distributions of *OR_DM-LTF_* by scenario.

The direction of the error was primarily determined by the exposure classification parameters. In the four scenarios where workers with the highest exposure were more likely than those never-exposed to be misclassified as alive, the adjusted OR moved away from the null (*i.e.*, the crude OR was biased toward the null). In contrast, when the never-exposed group had a greater proportion misclassified as non-cases than the highest exposure group (Scenarios 1, 3, 5, and 7), adjustment for study bias due to loss to follow-up resulted in a shift of the *OR_DM-LTF_* frequency distributions toward the null, lessening the observed effect for the exposure-disease relationship (Figure 4).

In all the scenarios we examined, uncertainty about the amount of disease misclassification due to assuming that lost subjects were alive resulted in uncertainty about the magnitude of the TCDD-IHD association estimate.

## 4. Discussion

Here we demonstrated that losses to follow-up in a historical cohort mortality study can cause disease misclassification. This source of disease misclassification is different from the misclassification that may result from using death certificate or other mortality data and is unique to historical cohort mortality studies. Currently, methods using person-time to conduct probabilistic uncertainty analyses have not been published. As such, one must use either the incidence proportion ratio or the odds ratio as the estimator when conducting bias analyses of these studies. When using either of these measures, however, those lost to follow-up are consequentially counted as alive even though some may have died from the disease of interest. For both measures, study subjects contribute to the denominator but have no opportunity to contribute to the numerator. The only way to prevent this would be to exclude anyone who was lost, but this creates other potential problems and has never been an accepted method for addressing losses to follow-up.

Since we had no reason to assume that disease misclassification caused by losses to follow-up would not affect the association measure for occupational exposure to TCDD and IHD mortality, we developed a probabilistic bias analysis method to adjust for this source of systematic error using Monte Carlo simulations. In developing this method, we encountered several issues that challenged the implementation of our approach. First, very little information was available from the original study [13] on how losses were distributed across the exposure categories. This would likely be easier to address when the exposure variable is dichotomous, but can become particularly complicated with multi-level exposures. We believed that the best method of making the process more manageable was to divide it into multiple steps, which also allowed us to check the accuracy of our process at different points. In addition, it was necessary to calculate the number of living subjects in each exposure group and set this as the maximum distribution value when the total number for a given step exceeded the number of living that could have been misclassified as alive. For example, 148 workers were categorized as non-cases with exposure ≥2085.8 ppt TCDD-mo, but only 112 of these employees were classified as alive at the end of the study. The other 36 subjects died of causes other than IHD and could not have been potentially misclassified as alive due to losses to follow-up. Accordingly, we used the IF function in Excel to set the distribution maximum for the number of IHD deaths in the exposure group “≥2085.8 ppt TCDD-mo” equal to 112 when the total number of “ever-exposed” that were potentially misclassified as alive (*i.e.*, died of IHD) was greater than 112. Otherwise, the maximum value for this parameter equaled the number of IHD deaths among those lost that were “ever-exposed”. We also addressed a variety of concerns with regard to the simulation software used and the distributions available. For the number of all-cause deaths among losses, we chose a negative binomial distribution because it was discrete and provided the flexibility we needed to describe the distribution for this parameter. Unfortunately, the negative binomial distribution is determined by probability and shape, which are not easily varied, so it was not an ideal distribution for the remaining three classification parameters—each of which are dependent on the preceding step. We initially considered the Poisson distribution because the peak (λ) could be determined based on the trial value of another classification parameter. However, this distribution was not as flexible as we had hoped. For example, if the number of all-cause deaths among losses was 104, the distribution for the total number of IHD deaths would need to peak at 21, assuming a proportion of 20.4%, and have a range of 0 to 104. With a Poisson distribution, the probability of the simulation choosing a count less than 9 or greater than 37 was zero, effectively excluding the majority of potential values. Rather, we chose to use the continuous BetaPERT distribution—which is renormalized over a finite range other than (0,1), re-parameterized by the minimum, maximum, and mode [15], and very flexible—and truncate the selected trial values to whole numbers. Comparison of the adjusted odds ratios and error terms estimated with and without use of the TRUNC function demonstrated any differences were negligible. Last, it should be noted that the simulation software we used would suspend a run anytime zero was selected as a trial value for the first two parameters or when the total number “ever-exposed” that were potentially misclassified as alive was zero. To address this, we used the IF function in Excel to set the subsequent classification parameter values to zero when any of the described conditions occurred (e.g., if a trial value for the total number of IHD deaths among losses was zero, then all successive parameter values for that trial would be zero). We believe this is a superior alternative to excluding these trials.

In the example described here, we demonstrated that the magnitude of the error from disease misclassification due to loss to follow-up can be substantial. The certainty intervals for *OR_DM-LTF_* in the simulations for this bias analysis were quite wide, with the distance between the upper and lower bounds ranging from 2.67 to 9.27. While the exposure parameters (“IHD deaths: never-exposed” and “IHD deaths: ≥2085.8 ppt-mo”) were the main determinants of the location of the geometric mean adjusted odds ratios, the width of the certainty intervals was likely influenced more by the degree of misclassification.

By using a probabilistic method that specifies a range of values for the classification parameters under a variety of thoughtfully constructed scenarios, our approach allows one to estimate intervals that may better represent the level of uncertainty from systematic study error in a given exposure-disease relationship. Compared to other techniques such as simple sensitivity analysis and inverse probability weighting (IPW), use of probabilistic bias analysis may be more advantageous because it does not rely on conditional weighting estimates or single sensitivity and specificity values. One of the primary limitations of IPW is that the model estimating the weights is built as a function of subject characteristics (e.g., age, gender). Anything that might be related to follow-up should be part of the model building process. Yet, when characteristics that may be predictive of follow-up are not measured or collected as part of the study, use of this method may actually introduce bias. Our method, however, allows one to use external data to adjust for any characteristic—whether or not measured as part of the initial study—that may affect follow-up or the outcome of interest.

Our primary objectives were to illustrate how to adjust an odds ratio for disease misclassification resulting from losses to follow-up in a historical cohort mortality study and to evaluate the effect of this source of error. It is likely, therefore, that uncertainty about the magnitude of other study limitations; for example, exposure misclassification would further increase the uncertainty about the TCDD-IHD association. Additionally, the method described here is contingent on the distributions constructed for each of the classification parameters, with the usefulness of the results limited by the accuracy of those distributions. For example, we used New Zealand mortality data from 2008, which assumes the proportion of deaths from IHD was constant over the entire study period evaluated by McBride *et al.* [13]. However, the New Zealand Ministry of Health reported that the percentage of IHD deaths declined over the 1980–2008 time frame [14] and we, therefore, may have underestimated the number lost to follow-up that died from IHD and the level of uncertainty.

## 5. Conclusions

In summary, our method can be employed to quantify and adjust for disease misclassification due to losses to follow-up in any historical cohort mortality study with a defined polytomous exposure variable. Use of our probabilistic bias method to adjust for this source of systematic error may well be a considerable improvement over the standard conjecture that, most often, incorrectly assumes such error would be non-differential and have little or no effect on the observed exposure-disease relationship.
